# Obituary

**Published:** 1851-10

**Authors:** 


OBITUARY.

We regret to hear of the death of Dr. William W. Tyler, which took place on the 27th of August, near Columbia, Tenn., of typhoid fever. Dr. T. was a young physician of great worth and promise, and had just entered upon the practice of his profession with bright prospects of success and usefulness.

